# The anterior cingulate cortex is necessary for forming prosocial preferences from vicarious reinforcement in monkeys

**DOI:** 10.1371/journal.pbio.3000677

**Published:** 2020-06-12

**Authors:** Benjamin M. Basile, Jamie L. Schafroth, Chloe L. Karaskiewicz, Steve W. C. Chang, Elisabeth A. Murray

**Affiliations:** 1 Section on the Neurobiology of Learning and Memory, Laboratory of Neuropsychology, National Institute of Mental Health, National Institutes of Health, Bethesda, Maryland, United States of America; 2 Department of Psychology, Yale University, New Haven, Connecticut, United States of America; 3 Department of Neuroscience, Yale School of Medicine, New Haven, Connecticut, United States of America; 4 Kavli Institute for Neuroscience, Yale School of Medicine, New Haven, Connecticut, United States of America

## Abstract

A key feature of most social relationships is that we like seeing good things happen to others. Research has implicated the anterior cingulate cortex (ACC) in attaching value to social outcomes. For example, single neurons in macaque ACC selectively code reward delivery to the self, a partner, both monkeys, or neither monkey. Here, we assessed whether the ACC’s contribution to social cognition is causal by testing rhesus monkeys (*Macaca mulatta*) on a vicarious reinforcement task before and after they sustained ACC lesions. Prior to surgery, actors learned that 3 different visual cues mapped onto 3 distinct reward outcomes: to self (“Self”), to the other monkey (“Other”), or to neither monkey (“Neither”). On each trial, actors saw a cue that predicted one of the 3 juice offers and could accept the offer by making a saccade to a peripheral target or reject the offer by breaking fixation. Preoperatively, all 6 actors displayed prosocial preferences, indicated by their greater tendency to give reward to Other relative to Neither. Half then received selective, bilateral, excitotoxic lesions of the ACC, and the other half served as unoperated controls. After surgery, all monkeys retained the social preferences they had demonstrated with the preoperatively learned cues, but this preference was reduced in the monkeys with ACC lesions. Critically, none of the monkeys in the ACC lesion group acquired social preferences with a new set of cues introduced after surgery. These data indicate that the primate ACC is necessary for acquisition of prosocial preferences from vicarious reinforcement.

## Abstract 2

Why do we like seeing good things happen to others? A study of rhesus monkeys playing a juice donation game demonstrates that the anterior cingulate cortex is necessary for monkeys to acquire normal prosocial preferences.

## Introduction

Neuroscientists are just beginning to understand how the brain assigns value to social outcomes and translates that value to stable social preferences. A growing body of literature has implicated the medial frontal cortex (MFC), and specifically the anterior cingulate cortex (ACC), in processing social value [1]. In humans, a meta-analysis of functional magnetic resonance imaging (fMRI) studies has shown broad overlap in the MFC of activations produced by reward judgements and activations produced by social judgements [2]. Brain activations from fMRI in the ACC signal predictions about social approval [3], ownership by oneself or another [4], the value of rewards given to others [5,6], the probability that rewards will go to another person [7], and the prediction error after observing the outcome of others’ choices [6]. In clinical populations, the ACC has been identified as part of a broader network that shows abnormal fMRI activation in cases of psychopathy [8–11] and autism spectrum disorder [12], diagnoses that differ in many ways but that both show social processing abnormalities. The ACC of individuals with autism spectrum disorder shows altered cytoarchitecture [13], decreased axon guidance proteins [14], an abnormal bimodal distribution of Von Economo neurons [13,15], and altered activity in response to social prediction errors [16]. Damage to medial regions including the ACC produces social deficits that include trouble identifying or noticing others’ emotions [8,17].

In rodents, ACC activity tracks both experienced and observed pain [18]. Deactivating the ACC reduces rats’ responses to seeing others get shocked [18,19], impairs observational fear learning [20], and increases rats’ willingness to deliver a shock to a social partner [21]. Excitotoxic lesions of the ACC decreased rats’ social behavior without affecting their aggressive behavior [22]. Thus, the social functions of the ACC seem to be broadly conserved across taxa.

In nonhuman primates, neurons in the dorsal convexity of the MFC selectively code reward for either an actor monkey or a partner monkey in a Pavlovian cued paradigm [23]. In addition, neurons in the monkey dorsal ACC code the predicted behavior of a conspecific while playing an iterative cooperative game based on the history of the pair’s decisions [24]. Furthermore, resting state functional connectivity between the ACC gyrus and superior temporal sulcus in monkeys correlates with the size of a monkey’s social network [25], suggesting that ACC gyrus may assign value to social information typically processed by cortex in the superior temporal sulcus, such as faces. Finally, relative to controls, monkeys with ablations of the ACC gyrus show a reduced latency to reach toward social stimuli to obtain food [26], suggesting that this region is necessary for the processing of social cues.

In the paradigm most related to the current study, single-unit activity was recorded from neurons in the ACC gyrus, ACC sulcus, and orbital frontal cortex while monkeys performed a vicarious reinforcement task [27]. Actor monkeys chose between cues that predicted juice outcomes for themselves and a partner monkey. Monkeys showed context-dependent behavioral preferences—prosocial when choosing between cues that predicted reward to other or reward to neither and antisocial when choosing between cues that predicted reward to self or reward to both. Importantly, these preferences went away when the partner monkey was replaced with a juice collection jar. Thus, the pattern of findings supports the idea that vicarious reinforcement from others’ reward outcome is important for the observed prosocial preference [27]. Researchers found that neurons in all 3 areas were active in relation to distinct social decision outcomes, but the largest proportion of neurons selective for others’ reward outcome was located in the gyrus of the ACC [28]. Moreover, neurons in the gyrus of the ACC and the basolateral amygdala exhibit frequency-specific coordination that is enhanced for prosocial decisions but suppressed for antisocial decisions [29]. Taken together, the primate ACC seems centrally involved in processing social value. However, it remains unclear whether this involvement is causal, i.e., whether the ACC is required for vicarious reinforcement to produce social preferences.

**Fig 1:**
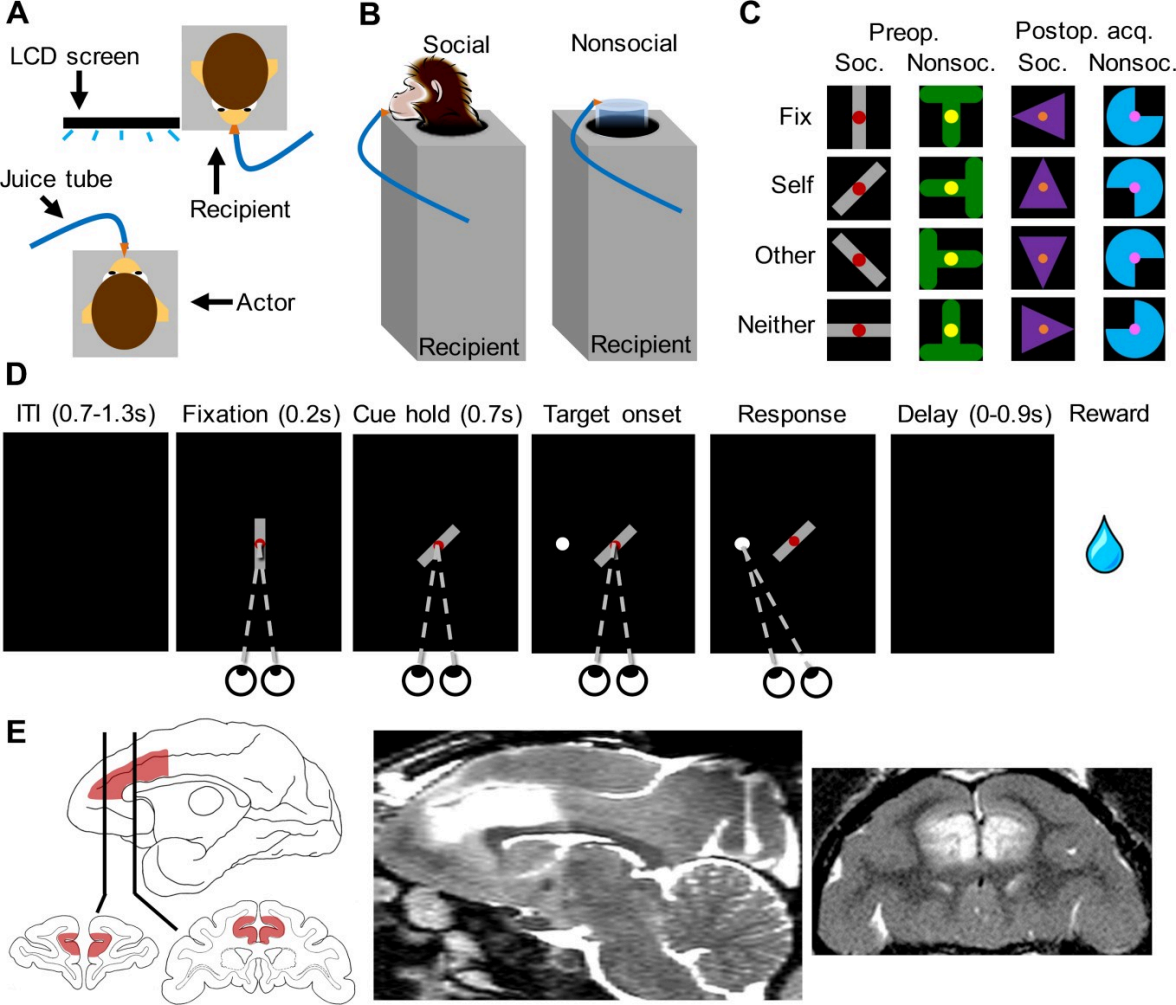
Monkeys completed a social vicarious reinforcement test before and after selective ACC damage. (A) Top-down schematic of the test arrangement with the actor monkey facing an LCD screen next to a recipient. (B) Schematic side view of juice delivery to recipient or juice collection cylinder in Social and Nonsocial sessions. (C) Stimuli used in the Social and Nonsocial sessions in the preoperative test, postoperative retention test, and postoperative acquisition test. The cues used for fixation were rotated to create the 3 reward conditions. (D) Schematic of the trial progression in a Social session in which the stimulus signals that “reward to self” is on offer. If the monkey completed the saccade to the peripheral target, the reward condition on offer for that trial was implemented. Note that the white peripheral saccade target appeared equally often in one of 8 locations equidistant from the center. (E) Diagram of the intended lesion in sagittal and coronal views (left) and postoperative edema (white hypersignal) observed in a T2-weighted MR scan in one monkey in the ACC lesion group. MR images from all monkeys with ACC lesions available in S1 Fig. ACC, anterior cingulate cortex; ITI, intertrial interval; LCD, liquid crystal display; MR, magnetic resonance.

In the current study, we evaluated whether the ACC was necessary for the prosocial preferences exhibited in monkeys’ social decisions by testing rhesus monkeys (*M*. *mulatta*) on a vicarious reinforcement task before and after they received selective, excitotoxic lesions of the ACC. In this task, an actor monkey faced a computer screen, and a familiar recipient monkey sat next to the screen facing the actor (Fig 1A). A visual cue on the screen rotated to signal juice outcome: reward to self (“Self”), reward to the other monkey (“Other”), or reward to neither monkey (“Neither”) (Fig 1C). On each trial, the actor had to choose whether to accept the juice offer by making a saccade to a peripheral target or reject the offer by breaking fixation (Fig 1D). On Nonsocial control sessions, the recipient was replaced with a graduated cylinder while all the other aspects of the task were kept the same (Fig 1B). To focus on the previously reported prosocial preferences, our critical comparison is the proportion of trials monkeys completed in the Other and Neither conditions. In a previous study of the autonomic correlates of vicarious reinforcement, we showed that monkeys had a reliable prosocial preference for accepting more Other offers than Neither offers and that pupils were paradoxically larger in anticipation of the less preferred Neither trials than the more preferred Other trials [30]. Here, we evaluated the degree to which this prosocial preference and autonomic arousal changed as a result of selective ACC damage (Fig 1E). We first evaluated whether monkeys would retain their baseline prosocial preferences when they were tested with the same cues after surgery or rest, and then evaluated whether they would acquire those same prosocial preferences when tested with novel cues.

## Results

**Fig 2:**
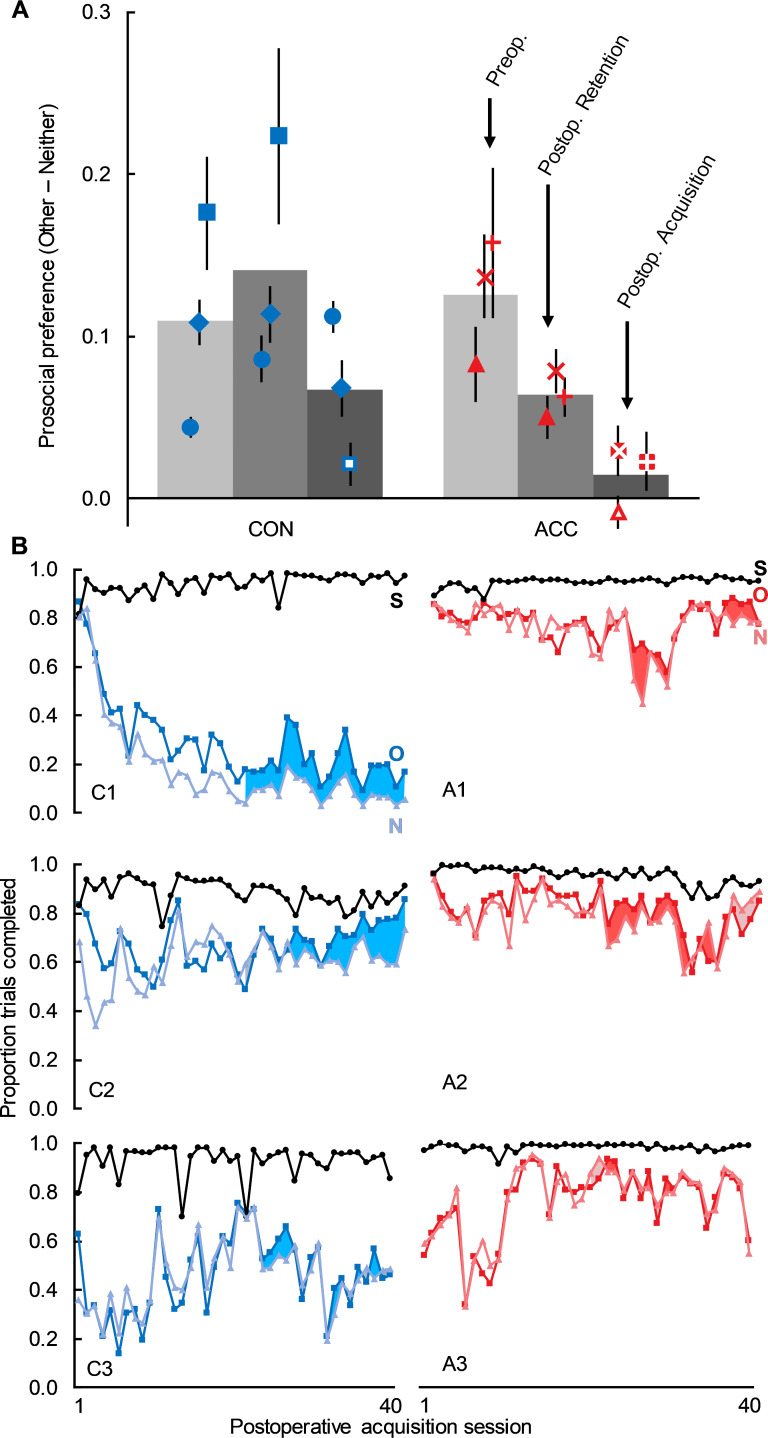
ACC damage reduced preoperatively learned social preferences and eliminated formation of postoperative social preferences. (A) Prosocial preference measured as the proportion of Other trials completed minus the proportion of Neither trials completed. Positive values indicate prosocial preferences, and negative values indicate antisocial preferences. Bars show group means, and points show scores of individual monkeys (±SEM) for the preoperative baseline preference (light grey), the postoperative retention test with the preoperatively learned cues (medium grey), and the postoperative acquisition test with novel cues (dark grey). Closed symbols indicate monkeys with a significant prosocial preference, and open symbols indicate monkeys with no significant preference. (B) Trial completion rates for postoperative acquisition sessions. Proportion of trials completed for all 40 acquisition sessions as a function of trial type. Black circles = Self. Dark squares = Other. Light triangles = Neither. Each panel shows scores of one monkey, with the control monkeys on the left (C1–C3) and the lesion monkeys on the right (A1–A3). The final 20 sessions (i.e., sessions 21–40) correspond to the data depicted in panel A; the difference between Other and Neither is highlighted for those sessions. Underlying data can be found in S1 Data. ACC, anterior cingulate cortex; CON, control group.

Preoperatively, all 6 actor monkeys showed a reliable prosocial preference for Other trials over Neither trials [30], and the magnitude of this preference did not differ between the groups designated to serve as controls or receive surgery (Fig 2A; t_3_ = 0.37, *p* = 0.735).

First, we examined the effect of the ACC lesion on retention of prosocial preferences. All actor monkeys received either a postoperative (ACC group) or post rest (unoperated controls) retention test with the preoperatively learned cues. As expected, all 3 controls exhibited significant prosocial preferences for Other over Neither (Monkey C1: t_19_ = 5.93, *p* < 0.001; Monkey C2: t_19_ = 6.53, *p* < 0.001; Monkey C3: t_19_ = 4.10, *p* < 0.001). The magnitude of the prosocial preferences did not differ between the baseline performance and retention test (baseline Other-Neither = 0.11; retention Other-Neither = 0.14; t_2_ = 2.36, *p* = 0.142). As a group, monkeys with ACC lesions showed a trend towards a reduced prosocial preference (Fig 2A; baseline Other-Neither = 0.13, postoperative retention Other-Neither = 0.06; t_3_ = 3.43, *p* = 0.076). But this reduced preference was still significantly above indifference in each individual monkey (Fig 2A; Monkey A1: t_19_ = 3.78, *p* = 0.001; Monkey A2: t_19_ = 5.74, *p* < 0.001; Monkey A3: t_19_ = 5.21, *p* < 0.001).

Second, we examined the effect of ACC lesions on the acquisition of prosocial preferences. In the postoperative acquisition test with novel cues, 2 of the 3 control monkeys reacquired significant prosocial preferences (Fig 2A and 2B; C1: t_19_ = 10.92, *p* < 0.001; C2: t_19_ = 3.90, *p* < 0.001; C3: t_19_ = 1.59, *p* = 0.127), but none of the monkeys with ACC damage acquired significant prosocial preferences (Fig 2A and 2B; A1: t_19_ = 0.83, *p* = 0.415; A2: t_19_ = 1.89, *p* = 0.075; A3: t_19_ = 1.25, *p* = 0.228).

Importantly, the lack of prosocial preferences in the ACC lesion group was not due to a general learning deficit, as all monkeys learned to prefer Self trials more than Other and Neither trials by the end of the first postoperative acquisition session, and trial completion rates for Self trials remained near ceiling throughout the acquisition sessions (mean ± SEM; Control: 0.93 ± 0.008; ACC: 0.97 ± 0.002). Furthermore, the monkeys were given extensive opportunity (40 sessions of the Social condition) to acquire and exhibit prosocial preferences, had they been present. Original acquisition of the prosocial preference took place within approximately 20 sessions (mean = 28.3, median = 20.0) and was often apparent in individual monkeys well before they had completed the minimum required 20 preoperative Social sessions.

In intact (i.e., unoperated) monkeys, preference for Other is dependent on the presence of the partner monkey; baseline prosocial preferences are absent in the Nonsocial condition [30]. In the Nonsocial sessions of the retention test of this study, only one monkey (C3) showed a numerical preference for giving juice to the collection jar, but this was not significant (S2 Fig; t_19_ = 0.87, *p* = 0.398). For Nonsocial sessions of the acquisition test, no control monkey showed a numerical prosocial preference (S2 Fig). Unexpectedly, 2 of the monkeys in the ACC lesion group showed weak but reliable tendencies to give juice to the collection jar, and the third showed a nonsignificant trend in the same direction (S2 Fig; A1: t_19_ = 3.59, *p* = 0.002; A2: t_19_ = 3.12, *p* = 0.006; A3: t_19_ = 2.01, *p* = 0.059). This is especially striking considering that none of these monkeys showed a prosocial preference during the Social sessions. Future research will be needed to evaluate whether this is a case of behavioral facilitation following lesions (e.g., [31]).

**Fig 3:**
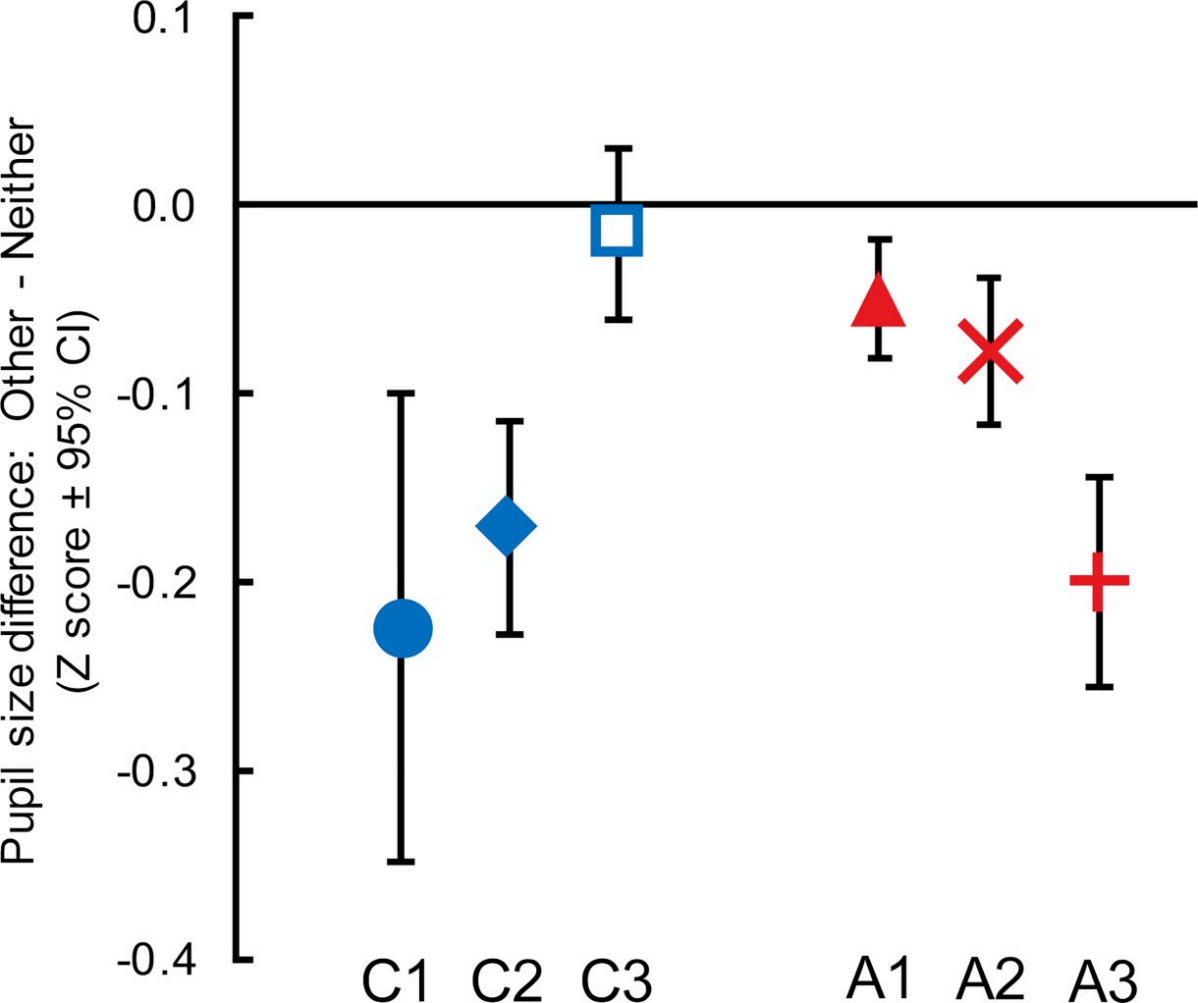
Monkeys without prosocial preferences after ACC lesions still showed pupil size differences on Other versus Neither trials. Difference in mean pupil change from baseline (Z score ± 95% CI) between Other and Neither trials during the 50-ms epoch fixating on the peripheral saccade target prior to juice delivery in Other and Neither trials. Positive values indicate larger pupils on Other trials, and negative values indicate larger pupils on Neither trials. Data are from the final 20 sessions of postoperative acquisition, Social condition only. Control monkeys: C1–C3; monkeys with ACC lesions: A1–A3. See also S3 Fig for the pupil widths in Other and Neither trials that went into this difference score. Underlying data can be found in S2 Data. ACC, anterior cingulate cortex.

Preoperatively, monkeys’ autonomic arousal was dissociated from their preferences, in that pupil size was larger for the less preferred Neither outcomes than for the more preferred Other outcomes [30]. Importantly, this difference in autonomic arousal between the Other versus Neither reward conditions was specific to the Social condition, as pupil size associated with delivering juice rewards to the juice collection jar did not differ from the Neither condition [30]. Postoperatively, pupil size remained dissociated from social preference. The 2 control monkeys who reacquired their previous prosocial preferences and all the monkeys with ACC damage still showed wider pupils for the Neither outcome relative to the Other outcome (Fig 3, S3 Fig). Notably, the only monkey that did not show a difference in pupillary response during postoperative acquisition sessions was also the one control monkey that did not reacquire his previous social preference (Monkey C3; Fig 3 and S3 Fig), suggesting that this monkey may have acquired social preference in a different way compared with the rest of the monkeys.

## Discussion

Monkeys with selective excitotoxic lesions of the ACC did not reacquire the prosocial preference they had exhibited preoperatively, suggesting that the ACC is critical for vicarious reinforcement necessary for developing the prosocial preference. Thus, the neural activity observed in the ACC during vicarious reinforcement [28] may underlie the social decisions in this task. This is consistent with the idea that the ACC, especially the gyrus, is a site where value signals are combined with social signals to produce social valuation based on information from others [1]. On this view, damage to the ACC prevents monkeys from attaching value to another monkey’s reward outcome, which, in turn, prevents learning from vicarious reinforcement in social decision-making and development of a stable prosocial preference. Notably, damage to ACC gyrus has been predicted to yield just this result: a reduced sensitivity to other’s rewards that manifests as a failure to maintain prosocial behaviors [32]. Our ACC lesions did not prevent monkeys from learning which cue predicts juice rewards to themselves, suggesting that the deficit is specific to assigning value to social information. Although a prior study causally implicated the ACC gyrus in the processing of social cues [26], the current study offers at least 2 specific advances. First, unlike the earlier study that used aspiration lesions, the monkeys in the present study received excitotoxic, fiber-sparing lesions. Thus, we can be confident that any behavioral effects of the lesions were due to loss of neurons in the sulcus and gyrus of the ACC and not to unintended damage to fibers of passage. Second, the present study specifically evaluated the role of ACC in mediating monkeys’ tendencies to give rewards to others (e.g., based on vicarious reinforcement). Accordingly, we conclude that the ACC is essential for integrating social context and reward cues.

Another major finding of the present study is that although ACC lesions disrupt monkeys’ social decision-making, evidenced by altered preference of social reward outcomes, they have no effect on autonomic arousal in anticipation of those same outcomes. This is surprising given that the ACC is strongly connected to the locus coeruleus [33,34], and locus coeruleus activity correlates with pupil size [35]. In addition, aspiration lesions of the neighboring subgenual ACC blunt the sustained pupil dilation in anticipation of primary reward [36]. Thus, we might expect that the ACC is part of a network that regulates autonomic arousal in anticipation of reward outcomes in social settings. The lack of effect of ACC lesions on autonomic arousal in this study indicates that the ACC is not critical for mediating autonomic responses in anticipation of social reward outcomes of the type used here.

One caveat about our interpretation that behavioral preference was dissociated from pupillary response is that it relies on the assumption that pupil dilation correlates with anticipated value, presumably because higher value choices produce more sympathetic autonomic arousal than lower value choices. This assumption is reasonable because several carefully conducted experiments have shown that pupil size scales with emotional arousal [37] and the value of primary reinforcement [36,38]. However, pupillary responses are complex [39], pupil size is affected by factors other than anticipated value [40,41], and even reliable value-related pupil changes are more nuanced than a simple model in which high anticipated value always produces large pupils [42]. Indeed, our own measures of pupil response during vicarious reinforcement demonstrate some of this nuance [30]. Regardless, our results suggest that the ACC is not a part of the neural circuitry responsible for producing the socially modulated pupil response seen during vicarious reinforcement.

In our retention test, we found that the prosocial preferences were reduced but still present after ACC lesions. This partial sparing demonstrates that social preferences, once formed, become partially independent of the ACC, especially the ventral sulcus and the gyrus to which most of the damage was localized (Fig 1E; S1 Fig). One possibility is that this spared preference reflects a habitual positive value for the stimulus itself rather than a value for the associated outcome. Under this model, the cue rotation is the conditioned stimulus (CS), the sight of a social partner receiving reward is the unconditioned stimulus (US), and the ACC damage prevents the CS from being further associated with the US, effectively putting the preference into extinction. This would explain both the blunted but significant preference to the preoperatively learned cues—because conditioned preferences persist during extinction—and the lack of the same preference postoperatively—because the new neutral stimulus cannot be paired with the US.

Previous work allows us to speculate about what mechanism underlying vicarious reinforcement might have been compromised in our monkeys with ACC damage. One possibility is that the damage interfered with processing of a social prediction error—the discrepancy between the expected and actual outcome for the other monkey. ACC activity in humans and monkeys tracks social prediction errors [6,16], and disrupting this prediction error may have interfered with learning during the acquisition sessions. Another intriguing possibility is that the damage interfered with outcome ownership assignment—the ability to associate an outcome with oneself or another. ACC activity in humans correlates with ownership assignment and prediction errors following ownership judgement [4], and the inability to associate juice outcomes with another monkey versus nobody—or, alternatively, to associate a visual cue with another monkey—might have led to the observed indifference. These explanations are not mutually exclusive; ACC lesions may have disrupted ownership prediction errors. More research will be needed to tease apart these explanations.

Although our study provides evidence for a causal contribution of the primate ACC to learning about social outcomes, it is not without limitations. First, we studied a total of 6 monkeys, and the size of our 2 groups is small (*n* = 3). Thus, the generalizability of our finding must be viewed cautiously. This also precludes potentially informative analyses of how factors like dominance rank or partner familiarity affects the observed lesion effect. Anecdotally, we note that our lesion group included 2 monkeys who were likely dominant to their recipient and 1 who was likely submissive to his recipient. The prosocial preferences in all 3 were affected by the lesion. Despite this limitation, when taken in the context of the findings from humans, rodents, and monkeys [1,2,4–9,11–14,17–21,23–26,28,29,34,43,44], our findings help further specify the role of the ACC in social cognition.

Prior work suggests that the locus of social valuation might be limited to the gyrus of the ACC, a region smaller than that targeted by our lesion. In the recording study that focused on the ACC gyrus and sulcal cortex, a greater proportion of ACC gyrus neurons, relative to neurons in the ACC sulcus or orbital frontal cortex, coded rewards to the other monkey [28]. Similarly, aspiration lesions of cortex on the ACC gyrus more consistently reduced latency to reach toward social stimuli than did aspiration lesions of cortex in the ACC sulcus [26]. Future studies should determine whether selective excitotoxic damage limited to the ACC gyrus produces the same impairment as did our ACC lesion in the current study, which included both gyral and sulcal portions of ACC.

Dysfunction within ACC and related circuits has been implicated in clinical diagnoses involving altered social processing, such as psychopathy and autism spectrum disorder. The body of work implicating the ACC in social processing disorders in humans [8–15,17], combined with neurophysiological and lesion studies suggesting a role for the ACC in social behavior in macaques [1,2,23–26,28,29,34] and the current data demonstrating a causal role of the ACC in vicarious reinforcement, can help guide future research into the specific computations performed in the ACC. Moreover, several studies in rodents have demonstrated specific and causal functions of the ACC in observational fear learning [20,43], the social responses to seeing others get shocked [18,19], and the willingness to deliver a shock to a social partner [21], suggesting an evolutionarily conserved role of the ACC in mediating social learning broadly [44]. A better understanding of the causal contributions of the ACC to social information processing would come from casting a broader net to interrogate social cognition. Future studies might evaluate not only vicarious reinforcement, as done here, but also natural social interactions [45,46], natural viewing preferences for social and nonsocial images [47,48], explicit social judgements [49], and related phenomena.

In summary, damage to the ACC disrupts social decision-making by blunting inherent prosocial preferences and abolishing the ability to acquire new prosocial preferences in monkeys performing the vicarious reinforcement task. These findings provide causal evidence linking the primate ACC to prosocial preferences mediated by vicarious reinforcement. Vicariously derived information underlies many social functions ranging from social learning to understanding others’ behaviors to taking others’ perspectives. These functions likely mediate evolutionary fitness in social species living in large groups. Our findings here endorse the view that the ACC is a necessary functional node in the primate brain that enables the emergence of complex social cognition in primates.

## Materials and methods

### Ethics statement

All procedures were reviewed and approved by the National Institute of Mental Health Animal Care and Use Committee, operating under PHS Animal Welfare Assurance Number D16-00602 to the NIH Intramural Research Program, and complied with the US law and regulations as described in the Institute of Laboratory Animal Research Guide for the Care and Use of Laboratory Animals [50]. All monkeys received a program of food and toy enrichment, overseen by a dedicated primate enrichment specialist. Monkeys were housed singly due to concerns about altered social processing following surgery leading to injury but had visual and auditory access to multiple conspecifics in the room. Monkeys received ad libitum food and were on a 12:12 light:dark cycle. Total daily fluid was controlled so that monkeys maintained good motivation in the test apparatus and good health. Weight, appearance, and behavior of monkeys was monitored daily by researchers in coordination with veterinary staff.

During surgery, blood pressure, respiratory rate, heart rate, temperature, blood oxygen saturation, and exhaled/inhaled CO_2_ were monitored by veterinary staff to ensure the health of the monkey. Postoperative recovery and analgesia were directed by veterinary staff and included dexamethasone (4 mg/ml, intramuscular [i.m.], 1.5 ml), cefazolin (330 mg/ml, 25 mg/kg, i.m.), ketoprofen (100 mg/ml, i.m., 0.1 ml–0.2 ml), and ibuprofen (100 mg, orally).

### Subjects

Nine adult male rhesus macaques (*M*. *mulatta*) participated in the experiment (mean age = 6.5 y), 6 as actor monkeys and 3 as recipient monkeys. Two actors were assigned to each dedicated recipient and housed directly across from that recipient. In these triads, one actor was randomly assigned to the control group and one to the lesion group. Thus, actors in both groups were equally familiar with their recipient, and recipient’s identity did not differ between groups. Prior to this study, we implanted each monkey with a titanium head post to allow head-restrained eye tracking [51] and shaped each monkey to perform a basic oculomotor saccade task.

### Apparatus and stimuli

We tested monkeys in pairs in a sound-attenuating chamber (Crist). Actors sat in a primate chair facing a computer monitor (22.86 cm wide × 30.48 cm tall) at a distance of approximately 54 cm. Recipients sat in a primate chair such that their head was immediately to the right of the monitor (actor’s view) and they faced over the actor’s right shoulder (Fig 1A). Monkeys could easily view each other but did not directly face each other as it might evoke aggression in rhesus macaques [52]. Both monkeys were head restrained during testing. A camera positioned at the lower right corner of the monitor tracked the actor’s eye position and pupil width. Juice (50:50 apple juice:water) was delivered via hidden tubing to one of 2 metal spouts positioned at the mouth of either the actor or recipient. Pressurized juice-delivery systems [53] were housed outside the chamber, and delivery was gated by solenoids housed in their own sound-attenuating box. This box effectively silenced the juice delivery, rendering it undetectable by 2 separate humans who performed forced-choice and yes-no detection tests (proportion correct = 50% and d’ = 0.0, respectively). In addition, a sound meter placed approximately 5 cm away from the box did not register any sound increase from rapid solenoid firing when the lid was closed (maximum sound level during juice delivery with sound-attenuating box open = 58.82 dB [±0.60], during delivery with box closed = 49.89 dB [±1.10], and not during delivery = 50.42 dB [±1.17]). Still, to rule out any contribution of the solenoid to monkey's behavior, we took 2 additional precautions. First, the sound-attenuating box housed a third dummy solenoid that fired on the Neither reward trials, and a recorded audio clip of a solenoid firing was played inside the monkey testing chamber on every completed trial regardless of reward outcome. Stimuli were 4 abstract shapes that could appear in one of 4 orientations to signal the start of the trial or one of the 3 juice offers (Fig 1C). One shape was used on preoperative Social sessions, one on the preoperative Nonsocial control sessions, one on postoperative Social sessions, and one on the postoperative Nonsocial sessions (Fig 1B).

### Behavioral procedures

Two monkeys participated in the task at a given time, one actor and one recipient. The 6 actors were matched with 3 dedicated recipients such that each recipient worked with 2 actors, actors always worked with the same recipient, and no actor ever served as recipient.

Each trial began with the onset of the fixation stimulus (Fig 1D). After an actor monkey acquired and held central fixation for 0.2 seconds, the stimulus was replaced with one of 3 alternative orientations that predicted one of 3 juice outcomes: Self, Other, or Neither. The Self trials delivered juice to the actor, the Other trials delivered juice to the recipient on Social sessions or the juice receptacle on Nonsocial sessions, and the Neither trials delivered no juice. To accept the juice offer, the actor monkey had to maintain fixation for an additional 0.7 seconds until a peripheral saccade target appeared in one of 8 equidistant locations, and then had to make a saccade to that target. After a random delay of 0.0–0.9 seconds, the signaled juice outcome was delivered, and the actor had an additional 1 second of free viewing time to observe the recipient. To reject the juice offer, the actor could abort fixation after the rotated cue appeared or fail to saccade to the peripheral target. Aborted trials were followed by a white screen that lasted 5 seconds and were repeated if the actor aborted before having seen the juice offer but not repeated if the actor had seen the juice offer. All trials were separated by a blank interval of 0.7–1.3 seconds. Actors worked for either 0.3 or 0.5 ml of juice per reward, depending on individual motivation, and recipients always received 0.5 ml of juice per reward. Juice volume per reward was held constant within any given session. The delivery times were calibrated such that juice delivery—or unfilled interval if it was a Neither offer—lasted the same duration for all 3 conditions. Juice offers were pseudo-randomly determined, with the constraints that half of the offers were Self to maintain motivation, there were an equal number of Other and Neither offers, and a given offer type could appear no more than 4 times in a row. Monkeys completed one 600-trial session per day. Nonsocial sessions were identical to Social sessions except for the use of a different stimulus and the presence of a juice receptacle instead of the recipient monkey. To balance for whether Social or Nonsocial conditions were experienced most recently prior to surgery, we ran conditions in blocks of 10 sessions with an ABBA or ABAB pattern, with half of monkeys assigned to each pattern.

Preoperative data comprised the last 20 sessions each for the Social and Nonsocial conditions performed by the monkeys before surgery or rest. After postoperative recovery or an equivalent period of rest for controls, all monkeys were given a retention test, again comprised of 20 sessions each of the Social and Nonsocial conditions, using the same cues used preoperatively. When the retention test had been completed, all monkeys were trained with a new set of cues to determine whether they acquired prosocial preferences via vicarious reinforcement. We initially tested monkeys for 20 acquisition sessions in each of the Social and Nonsocial conditions. Because the 3 monkeys with an ACC lesion and 1 control failed to acquire prosocial preferences, we trained monkeys for an additional 20 sessions of each condition to test whether they would learn with more experience. Data illustrated in Fig 2A come from those final 20 sessions, in which monkeys had had the most chance to learn. Postoperative testing used the same ABBA or ABAB block design as preoperative testing.

### Surgery

We gave each actor in the lesion group bilateral ibotenic acid lesions of the ACC, primarily targeting areas 24 and 32, including both banks of the anterior portion of the cingulate sulcus and the immediately subjacent cingulate gyrus. This covers the area in which neural correlates of vicarious reinforcement performance were previously found [28]. During aseptic surgery, monkeys were immobilized with ketamine (100 mg/ml, 10 mg/kg, i.m.) and then anesthetized with isoflurane gas (1%–3% to effect). Monkeys received mannitol (25%–20%, 30–37 ml, i.v., at 90–111 ml/h) to reduce brain swelling and allow easier access to midline structures. We opened the skin and fascia/galea and retracted the temporalis muscle. We then opened a bone flap extending bilaterally over the dorsal cranium. For each hemisphere, we reflected a semicircle of dura toward the midline and, with the aid of an operating microscope, used sulcal landmarks to visually identify the cortex targeted for injection of excitotoxins. The posterior boundary of the lesion was defined by an imaginary coronal plane through the spur of the arcuate sulcus, at the point where it intersected the posterior limit of the curved portion of the arcuate sulcus. The anterior boundary of the lesion was defined as an imaginary coronal plane through the rostral end of the cingulate sulcus. The dorsal boundary was the lip of the dorsal bank of the cingulate sulcus, and the ventral boundary was an imaginary line running from the middle of the genu of the corpus callosum forward until it reached the anterior boundary. In each hemisphere, we made approximately 71 handheld injections (range = 61 to 80; 1 μl/injection) of ibotenic acid (10 mg/ml; Sigma) with a Hamilton syringe. The injections were roughly 2 mm apart. The injections in both hemispheres were carried out in a single operation. When the series of injections had been completed, we repositioned the dura over both hemispheres, sewed on the bone flap, and then closed the muscle, fascia/galea, and skin in separate layers.

### Lesion assessment

Three to seven days after each surgery, we acquired T2-weighted MR scans to visualize edema secondary to injection of excitotoxins and to confirm successful injections. In vivo T2 MRI has been shown to accurately predict damage in the hippocampus [54,55] but to overestimate damage in the amygdala [56]. Thus, we first verified the excitotoxic lesion approach in the ACC with postmortem histological examination in one pilot monkey. We observed excellent correspondence between observed damage and in vivo MRI hypersignal. Damage included almost the entirety of the ACC gyrus, including the target area identified by Chang and colleagues [28], the majority of the ventral bank of ACC sulcus, and a minority of the dorsal bank of sulcus. For the 3 actor monkeys in the present study, all still participating in other studies, in vivo MRI showed that lesions were generally as intended (Fig 1; S1 Fig). Interestingly, we saw unexpected sparing in the dorsal bank of the cingulate sulcus in all cases. It is unclear why this is the case, but it will be informative to see whether other studies also observe similar sparing. In all cases, the MRI hypersignal overlapped the target area identified by Chang and colleagues [28].

### Data analysis

Completion rates of Other and Neither trials were compared using paired *t* tests. We analyzed both as a group and for each individual monkey across sessions. Pupil traces were smoothed with a zero-phase low-pass digital filter using the “filtfilt” function in MatLab (MathWorks, Inc.) to compensate for the fact that our data acquisition system records at higher frequency than is sent by the eye tracker. Outliers in which the value at a particular millisecond was more than 3 SD away from the median of all other trials of that same type in that session were removed. We normalized the data for each trial as a proportion change from the initial 50 ms of that trial during fixation. All pupil data were expressed as z values, as in previous investigations of pupil size [35,36] to control for individual differences in pupil dynamic range. Statistical analyses were run on the last 50 ms of fixation to the cue and on the 50 ms of hold on the peripheral saccade target. All tests were two tailed with an alpha of 0.05.

## Supporting information

S1 Fig Lesion extent was largely similar across monkeys. Example postoperative images from T2-weighted MR scans of the 3 actor monkeys. Numbers next to each hemisphere indicate the volume of ibotenic acid injected. Monkey A2 is the monkey depicted in Fig 1.
(TIFF)

S2 Fig Monkeys generally did not show prosocial preferences in the Nonsocial control condition. Prosocial preference—the proportion of completed Other trials minus the proportion of completed Neither trials—for the Nonsocial control sessions in which the recipient was replaced with a graduated cylinder. Positive values indicate prosocial preferences, and negative values indicate antisocial preferences. Bars show group means, and points show scores of individual monkeys (±SEM) for the preoperative baseline preference, the postoperative retention test with the preoperatively learned cues, and the postoperative acquisition test with novel cues. Compare and contrast with Fig 2A. Underlying data can be found in S1 Data.
(TIFF)

S3 Fig Monkeys without prosocial preferences after ACC lesions still showed pupil size differences on Other versus Neither trials. Mean pupil change from baseline (Z score ± 95% CI) during the 50-ms epoch fixating on the peripheral saccade target prior to juice delivery in Other and Neither trials. More positive values indicate larger pupils, and more negative values indicate narrower pupils. Data are from the final 20 sessions of postoperative acquisition, Social condition only. Top row: control monkeys (C1–C3). Bottom row: monkeys with ACC damage (A1–A3). The difference in pupil size change between Other and Neither conditions is depicted in Fig 3. Underlying data can be found in S2 Data.
(TIFF)

S1 Data Trial completion data related to Fig 2 and S2 Fig. See S1 Data Readme for more information.
(ZIP)

S2 Data Mean pupil change data related to Fig 3 and S3 Fig. See S2 Data Readme for more information.
(ZIP)

## Abbreviations

ACC: anterior cingulate cortex
CS: conditioned stimulus; fMRI, functional magnetic resonance imaging
i.m.: intramuscular
ITI: intertrial interval
LCD: liquid crystal display
MFC: medial frontal cortex
MR: magnetic resonance
US: unconditioned stimulus
