# Supplementary figures and images for: The anterior cingulate cortex is necessary for forming prosocial preferences from vicarious reinforcement in monkeys
Source: PLoS Biol. 2020 Jun 12;18(6):e3000677. doi: 10.1371/journal.pbio.3000677 (PMC7292358; doi:10.1371/journal.pbio.3000677)

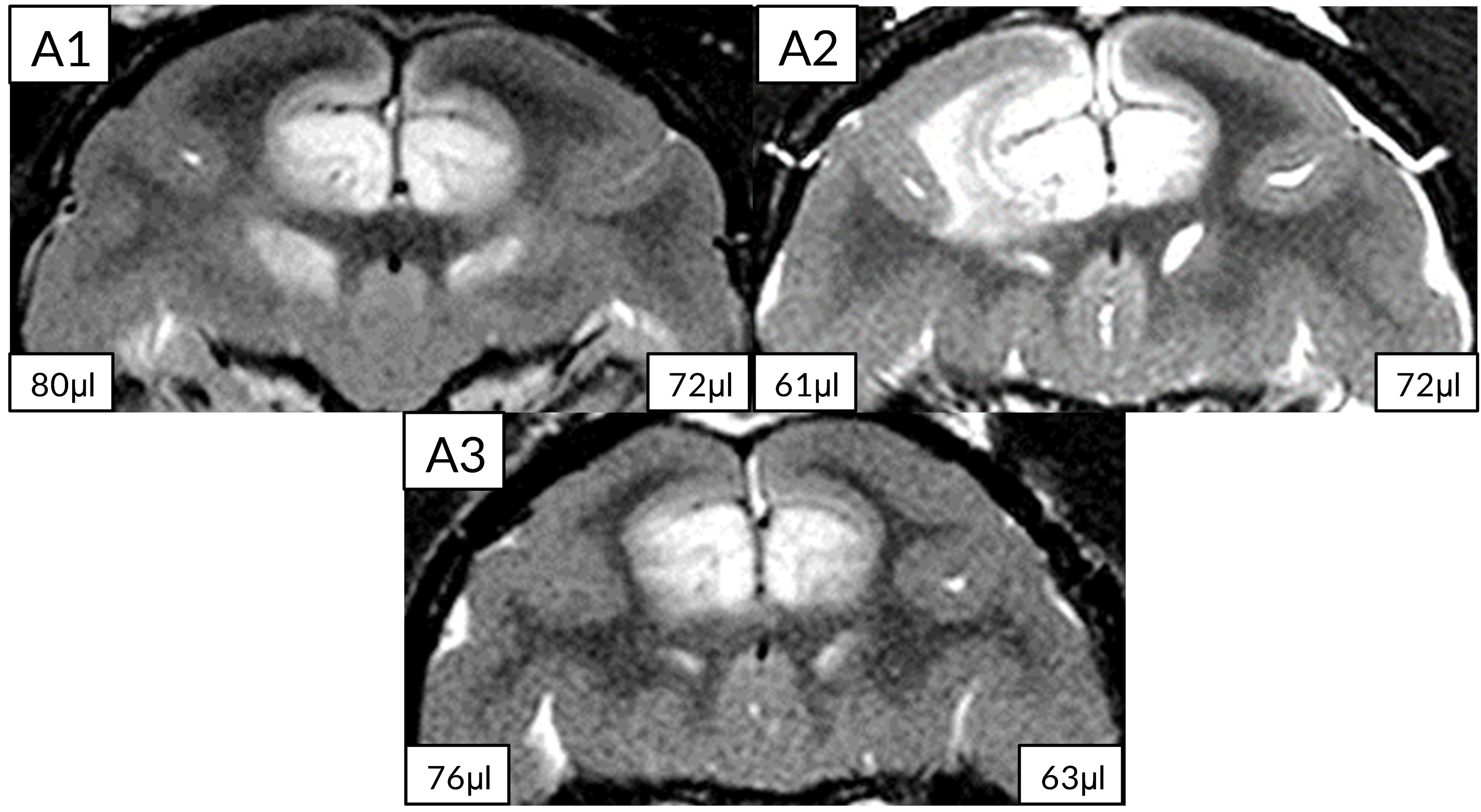

Supplement: S1 Fig — Example postoperative images from T2-weighted MR scans of the 3 actor monkeys. Numbers next to each hemisphere indicate the volume of ibotenic acid injected. Monkey A2 is the monkey depicted in Fig 1. (TIFF) [file pbio.3000677.s001.tiff]

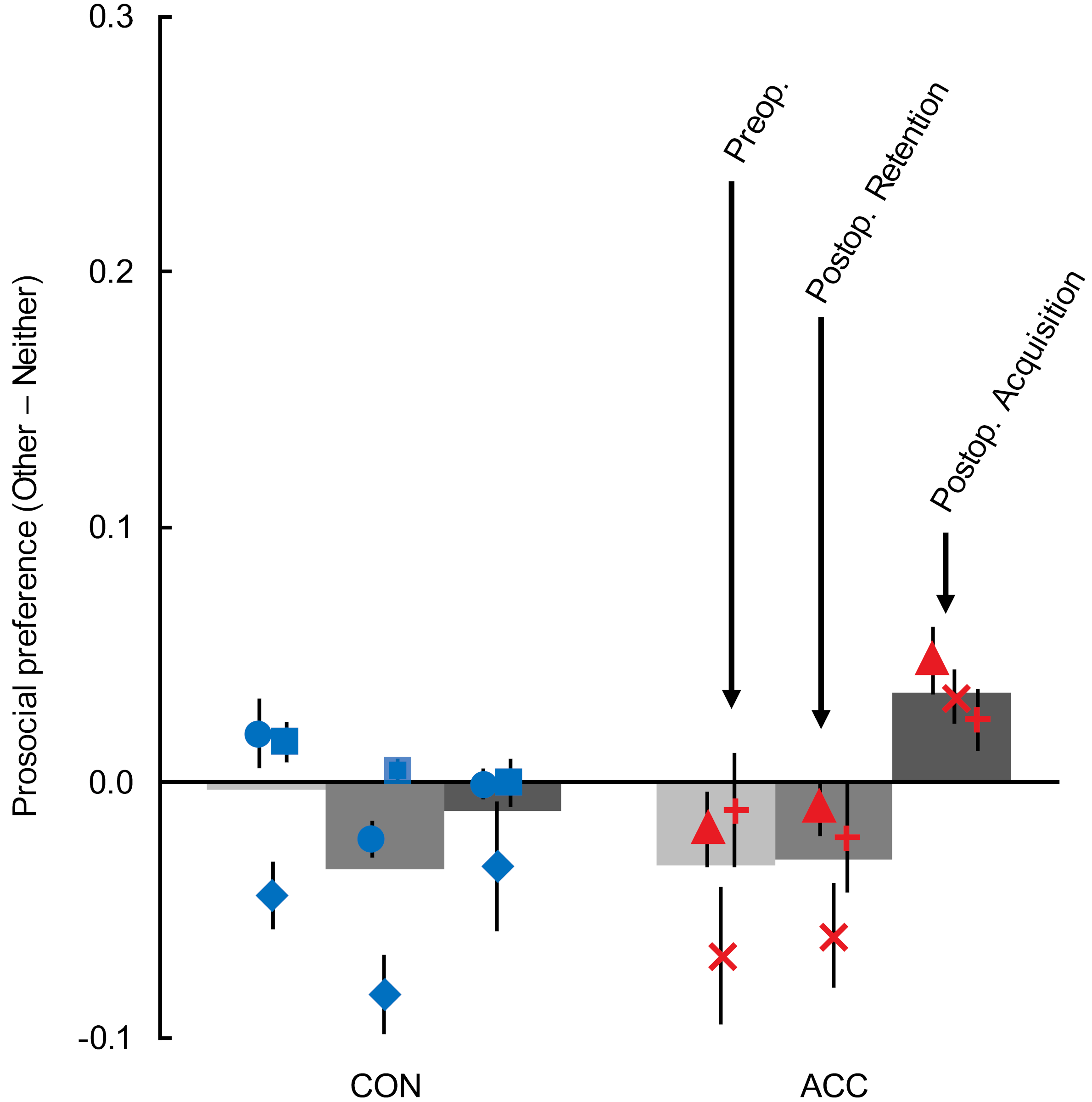

Supplement: S2 Fig — Prosocial preference—the proportion of completed Other trials minus the proportion of completed Neither trials—for the Nonsocial control sessions in which the recipient was replaced with a graduated cylinder. Positive values indicate prosocial preferences, and negative values indicate antisocial preferences. Bars show group means, and points show scores of individual monkeys (±SEM) for the preoperative baseline preference, the postoperative retention test with the preoperatively learned cues, and the postoperative acquisition test with novel cues. Compare and contrast with Fig 2A. Underlying data can be found in S1 Data. (TIFF) [file pbio.3000677.s002.tiff]

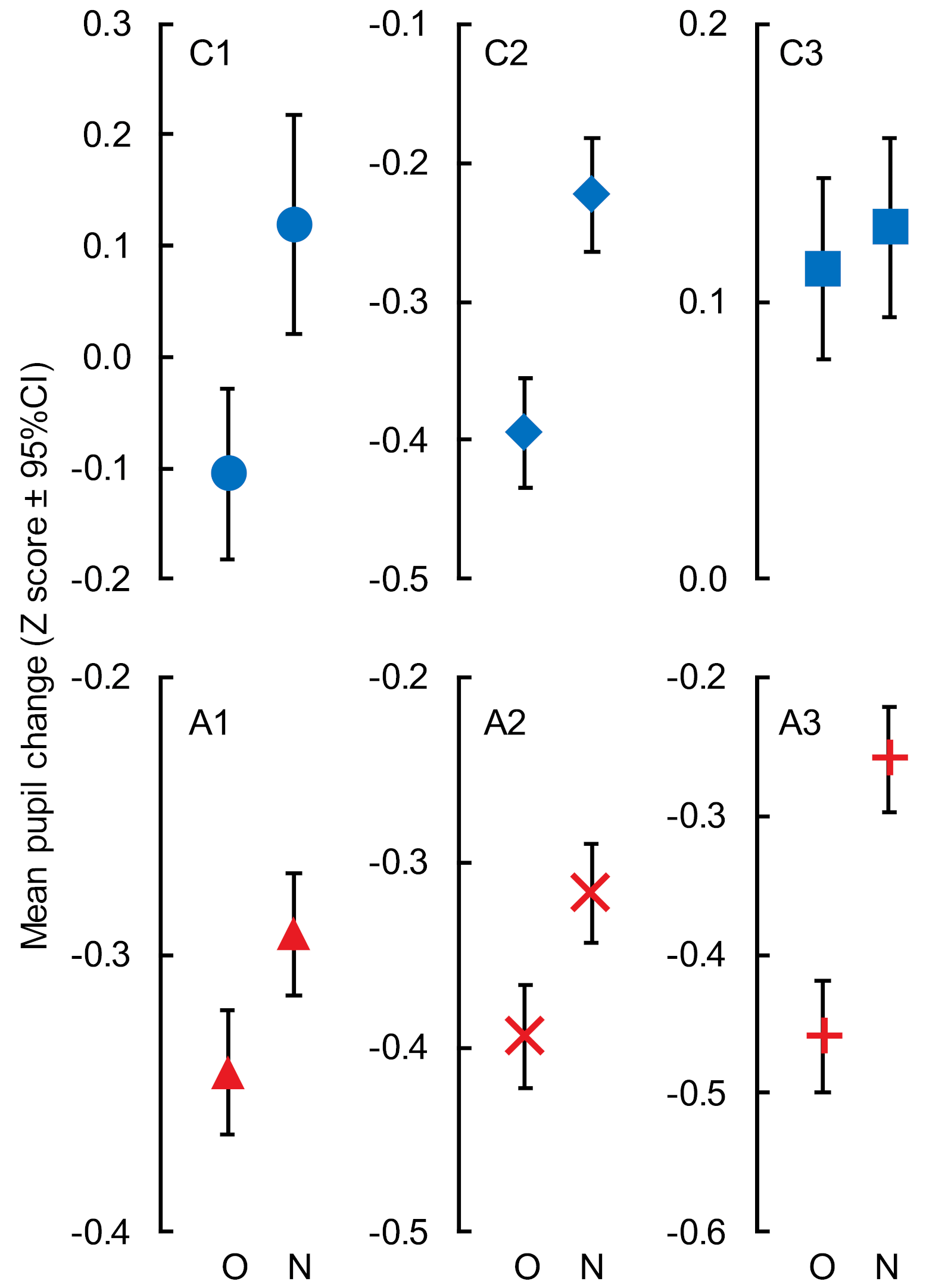

Supplement: S3 Fig — Mean pupil change from baseline (Z score ± 95% CI) during the 50-ms epoch fixating on the peripheral saccade target prior to juice delivery in Other and Neither trials. More positive values indicate larger pupils, and more negative values indicate narrower pupils. Data are from the final 20 sessions of postoperative acquisition, Social condition only. Top row: control monkeys (C1–C3). Bottom row: monkeys with ACC damage (A1–A3). The difference in pupil size change between Other and Neither conditions is depicted in Fig 3. Underlying data can be found in S2 Data. (TIFF) [file pbio.3000677.s003.tiff]
